# Voltage Affects the Dissociation Rate Constant of the m2 Muscarinic Receptor

**DOI:** 10.1371/journal.pone.0074354

**Published:** 2013-09-03

**Authors:** Yair Ben Chaim, Shimrit Bochnik, Itzchak Parnas, Hanna Parnas

**Affiliations:** 1 Department of Natural and Life Sciences, The Open University of Israel, Ra’anana, Israel; 2 Department of Neurobiology, Institute of Life Sciences, The Hebrew University, Jerusalem, Israel; Memorial Sloan Kettering Cancer Center, United States of America

## Abstract

G-protein coupled receptors (GPCRs) comprise the largest protein family and mediate the vast majority of signal transduction processes in the body. Until recently GPCRs were not considered to be voltage dependent. Newly it was shown for several GPCRs that the first step in GPCR activation, the binding of agonist to the receptor, is voltage sensitive: Voltage shifts the receptor between two states that differ in their binding affinity. Here we show that this shift involves the rate constant of dissociation. We used the m2 muscarinic receptor (m2R) a prototypical GPCR and measured directly the dissociation of [^3^H]ACh from m2R expressed Xenopus oocytes. We show, for the first time, that the voltage dependent change in affinity is implemented by voltage shifting the receptor between two states that differ in their rate constant of dissociation. Furthermore, we provide evidence that suggest that the above shift is achieved by voltage regulating the coupling of the GPCR to its G protein.

## Introduction

G-protein coupled receptors (GPCRs) are involved in the majority of signal transduction processes [1]. Binding of agonist to the receptor leads to stabilization of the active form of the receptor, which, when coupled to a trimeric G-protein, enhances GDP/GTP exchange within the α subunit of the G-protein. Subsequently, the GTP-bound form of the α subunit dissociates from the receptor as well as from the stable βγ dimer [2], [3]. Both GTP-bound α subunit and the released βγ dimer can activate intracellular effectors such as enzymes and ion channels.

Although the signaling pathway of GPCRs has been extensively studied, voltage sensitivity of GPCRs has only recently emerged as a novel signaling paradigm [4]–[16]. It was suggested that the function and affinity of many GPCRs are modulated by membrane potential. The most extensively studied voltage sensitive GPCR is the m2 muscarinic receptor (m2R). It was shown that both the affinity and the activity of the m2R are sensitive to changes in membrane potential [7], [10]. For this receptor it was further shown that depolarization induces movement of charges (analogous to gating currents in voltage gated ion channels) [6]. Finally, it was directly shown that the charge movement causes a conformational change in the receptor’s binding site [8], and corollary the affinity of the receptor toward its agonist was modified.

A relevant question at this point is whether the change in affinity is implemented via the rate constant of dissociation. Although some indirect evidence suggest that this might be the case [12], [16], further study is required.

To be able to address this question, it was necessary to do the measurements in living cells where membrane potential can be directly controlled. To achieve this goal, Xenopus oocytes were used. This heterologous expression system has been widely used to study biophysical properties of ion channels and receptors [17], and is ideal for the present research because the oocyte can express high levels of the receptor and hence ensure reliable measurements of specific binding of ligand to the receptors. Two independent approaches were taken: The dissociation rate constant of labeled ACh from the m2R was measured directly, to our knowledge for the first time, in individual, intact m2R-expressed Xenopus oocytes. This was done using a fast-washout protocol employed by us previously [6]–[8], [12], [13]. Second, we corroborated our direct measurements of dissociation of ACh by measuring deactivation of m2R induced G-protein activated inward rectifier K^+^ (GIRK) currents [18]. Under our experimental conditions the deactivation rate of m2R induced GIRK currents is determined by the dissociation rate of the agonist from the receptor.

Employing these two approaches, we found that the depolarization induced change in affinity of the m2R toward ACh is caused by depolarization shifting the m2R from a state with low dissociation rate constant (high affinity state) to a state with high dissociation rate constant (low affinity state). We further designed experiments to unravel the mechanism that underlies this voltage dependent shift between these states. Our results support the notion that membrane potential affects the coupling of the receptor to the G-protein, and that this coupling in turn determines the rate constant of dissociation and consequently the affinity state.

## Materials and Methods

### Ethics Statement

All experimental procedures used in this study were approved by the Hebrew University’s Animal Care and Use Committee (Ethical approval number NS-11-12909-3).

### Preparation of cRNA and Oocytes

cDNA plasmids of the two subunits of the G-protein activated inward rectifying K^+^ channel (GIRK) (GIRK1 and GIRK2), the m2R, and the m1R were linearized with the appropriate restriction enzymes. m2R mutants were prepared using QuikChange II Site-Directed Mutagenesis Kit (Stratagene, La Jolla, CA, USA) [6], [8].

The linearized plasmids were transcribed in vitro using a standard procedure [19].

*Xenopus laevis* oocytes were isolated and incubated in NDE96 solution composed of ND96 (in mM, 96 NaCl, 2 KCl, 1 CaCl2, 1 MgCl2, 5 Hepes, pH adjusted to 7.5 with NaOH), with the addition of 2.5 mM Na^+^ pyruvate, 100 U/ml penicillin and 100 µg/ml streptomycin (18). A day after their isolation, the oocytes were injected with the following cRNAs: m2R (2 ng/oocyte for the binding experiments and 200 pg/oocyte for the electrophysiological experiments); GIRK1 and GIRK2 (200 pg/oocyte for each); m1R (2 ng/oocytes).

Materials were purchased from Sigma Israel (Rehovot, Israel), unless stated otherwise.

### [^3^H]ACh Binding Experiments

**Figure 1:**
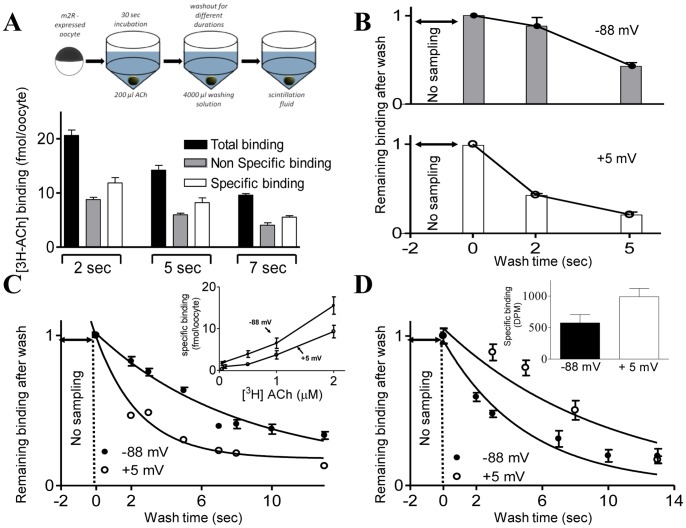
The dissociation rate of [^3^H]ACh from m2R-expressed oocytes is voltage dependent. (A, *top*) The measurement procedure (see Materials and Methods and [7]). (A, *bottom*) An example of data obtained from one experiment at resting membrane potential. Results after 2, 5 and 7 sec washout are shown (n = 9, 8 and 12 oocyte, respectively) (B) An example from one experiment at two membrane potentials as depicted. The data was normalized as follows: The binding of [^3^H]ACh was first measured after 2 sec washout in agonist free solution (initial sampling), and then after 2 and 5 sec of additional wash. (C) Collected data from a total of 9 experiments at resting potential (−88 mV) and under depolarization (+5 mV) fitted by an exponential decay. Each data point was normalized to the binding of the initial sampling and represents the average ± SEM of 16–59 oocytes (The SEM values at +5 mV are smaller than the symbols). *Inset*, results of binding measurements of [^3^H]ACh to m2R-expressed oocyte (Taken with permission from [7]). (D) Binding of [^3^H]ACh to m1R- expressed oocytes after different washout times, at two membrane potentials. Each data point was normalized to the binding of the initial sampling and represents the average ±SEM of 7 to 57 oocytes. *Inset*, results of binding measurements of [^3^H]ACh to m1R-expressed oocyte (Taken with permission from [7]).

The experiments were done following Ben-Chaim et al (2003). The oocyte was first dropped into a small chamber with 200 µl of either ND96 or high K^+^ solution (in mM: 2 NaCl, 96 KCl, 1 CaCl2, 1 MgCl2, 5 Hepes, with pH adjusted to 7.5 with KOH) containing a given concentration of labeled ACh ([^3^H]acetylcholine iodide, specific activity, 80 Ci/mmol, American Radiolabeled Chemicals, Inc., St. Louis, MO). The oocyte was incubated in this solution for 30 sec (60 sec for m1R-expressed oocytes), and rapidly dropped into a washing chamber filled with 4000 µl of ligand-free ND96 or high K^+^ solution. After the appropriate washing time the oocyte was rapidly removed from the washing chamber, and was dropped into a vial containing 3 ml of scintillation liquid. To avoid high non-specific binding and to avoid measurements inaccuracies, measurements have not been done with washout times shorter than 2 sec. The specific binding was measured as the difference between the total binding and the binding of labeled ACh to oocytes from the same batch at the same conditions but with no receptor expression (see Fig. 1A, *bottom* and [6], [7]).

For the PTX experiments, PTX protomer A (15ng/oocyte; list biological laboratories, Campbell, CA USA) was injected to oocytes 12–20 hours before the experiments. The uncoupling of G-protein from the m2R following PTX treatment was verified before each experiment by two electrode voltage clamp measurements of ACh induced GIRK currents [7].

### Current Measurements

The currents were measured 4–7 days after cRNA injection and were recorded using two electrode voltage clamp amplifier (Axoclamp 2B amplifier, Axon Instruments, Foster City, CA, USA). The oocyte was placed in the recording bath containing ND96 solution and was impaled with two electrodes pulled from 1.5 mm Clark capillaries (CEI, Pangboure, England). Both electrodes were filled with 100 mM KCl solution in order to prevent elevation of [K+]_in_ (The recording and the injecting electrode resistances were 15 and 2 MΩ, respectively). m2R mediated GIRK currents were measured in a 24 mM K^+^ solution (72 mM NaCl, 24 mM KCl, 1 mM CaCl2, 1 mM MgCl2, 5 mM Hepes, pH adjusted to 7.5 with KOH) pCLAMP8 software (Axon Instruments) was used for data acquisition.

### Data Analysis

τ_dis_ and τ_dec_ were measured by fitting a single exponential to the data. (See Results).

### Statistical Evaluation

Significance was checked by Student’s two tailed t-test. Results are given as mean ±SEM.

## Results

The m2R was shown to reside in one of two affinity (Kd) states; high, with Kd^H^, and low, with Kd^L^ [6], [7], [20]–[22]. Earlier studies demonstrated that depolarization does not affect the Kd itself, but rather shifts the m2R from high to low affinity state [6], [7]. In the context of the ternary complex model [23] the high and low affinity states reflect the receptor being coupled (high) or uncoupled (low) to its G-protein. We suggested that voltage exerts its effect by regulating the coupling of the receptor to its G-protein and thus its affinity state [6], [7].

Shift in affinity may be caused via the association rate constant (k_on_) and/or via the dissociation rate constant (k_off_) of the ligand. Here we confine our study to k_off_. To do so, we use Xenopus oocyte heterologous expression system. Two approaches were taken: (i) measuring the dissociation rate of labeled ACh and (ii) measuring the deactivation rate of m2R induced GIRK currents.

### Membrane Potential Affects the Dissociation Rate of ACh from the m2R

The dissociation of the labeled muscarinic ligand, [^3^H]ACh from m2R expressed oocytes was measured following different wash durations at two membrane potentials. (NMS, a common, high affinity, muscarinic antagonists could not be used because its affinity is voltage insensitive [21]). To achieve high specific binding, large amount of m2R cRNA was injected to the oocytes (2ng/oocyte), giving 15–20 fmole m2R molecules per oocyte, similar to the expression levels reported before [6], [7]. Changes in membrane potential were achieved by varying the [K^+^]_out_ (Controls demonstrating the lack of effect of [K^+^]_out_ per-se on the binding of ACh were done before [6], [7]). To increase the range of achievable membrane potentials, the two subunits of the GIRK channel, GIRK1 and GIRK2 were co-expressed in the oocytes [7]. The membrane potential was determined by standard intracellular microelectrodes for each oocyte batch.

The experimental protocol was as follows: An oocyte was incubated in [^3^H]ACh (250 nM, a concentration that yielded specific binding of ∼50% of the receptors at resting potential), for 30 sec (60 sec for the m1R, see below). Then, the oocyte was transferred to a washing vial containing either ND96 or high K^+^ solution. After different washing durations the oocyte was transferred to a vial containing scintillation fluid and the residual binding was measured ([7] and Materials and Methods).(Fig. 1A, *top*). Data obtained from one such experiment is shown in Fig. 1A, *bottom*. To extract the specific binding of [^3^H]ACh to the m2R, the binding of [^3^H]ACh to oocytes expressing the m2R and two subunits of the GIRK channel was first measured (total binding). Then, binding of the same concentration of ACh to oocytes expressing the GIRK channel but not the m2R was measured for each experimental membrane potential and for each washout time (non-specific binding). The specific binding was then determined by subtracting the average non-specific binding from the total binding of each oocyte.

Our technique does not enable measurements of dissociation of ligand at times shorter than 2 sec. Hence, we define the time that the initial sample was taken (2 sec after beginning of wash) as time 0 (Fig 1B–D). To be able to compare between different experiments and different membrane potentials, the remaining binding after washout longer than 2 sec is expressed as a fraction of the binding at time 0. The results of one experiment, at two membrane potentials, are depicted in Fig. 1B. It is seen that under depolarization (+5 mV) the dissociation rate of ACh is higher than that under resting potential (−88 mV). Cumulative results of experiments like the one shown in Fig. 1B (16–59 oocytes, 8 frogs) yielded the dissociation curves shown in Fig. 1C. These curves were fitted by exponentials from which the time constants of dissociation (τ_dis_) were extracted. It is seen that τ_dis_ is voltage dependent; it is 8.2 sec at −88 mV and 2.6 sec at +5 mV.

For interpretation of the results of Fig. 1C we recall the following. The m2R was shown to acquire two affinity states, high (R^H^, with Kd^H^) and low (R^L^, with Kd^L^) [6], [7], [20]–[22]. Depolarization was shown to shift the m2R from R^H^ to R^L^ [6], [7]. Thus, the measured Kd at any membrane potential (V) reflects the weighted Kd (Kd^W^) [6], as given in eq. 1
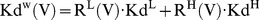
(1)and



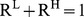



Note, that in eq. 1 the fraction of R^H^ and R^L^ varies with membrane potential [6], [7]. In contrast Kd^L^ and Kd^H^ are voltage independent [21], [22].

In view of the voltage dependence of τ_dis_ (Fig. 1C) and recalling that τ_dis_ = 1/k_off_ we can replace Kd^W^, Kd^L^ and Kd^H^ in eq. 1 by k_off_^W^, k_off_^L^ and k_off_^H^, respectively (eq. 2), or by the corresponding τ_dis_ (eq. 3)
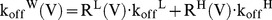
(2)




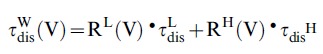
(3)


Our previous results indicate that at resting potential the majority of m2Rs reside in R^H^ [6]. Furthermore, measurements of the Kd of m2R in rat brain synaptosomes showed that Kd^L^ was larger than Kd^H^ by three orders of magnitude [21]. If follows that τ_dis_^L^ is expected to be 3 orders of magnitude smaller than τ_dis_^H^ Hence, for resting potential (V = V_rest_), eq. (2) may be simplified to become
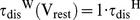
(3.1)


Thus, for −88 mV (Fig. 1C, filled circles) τ_dis_^W^≈ τ_dis_^H^ = 8.2 sec and k_off_^H^ (1/τ_dis_^H^) = 0.122 sec^−1^. At the higher depolarization (+5 mV) R^H^ is lower, and τ_dis_ accordingly is expected to decline as is indeed the case (Fig. 1C, open circles, τ_dis_ = 2.6 sec). Such reduction in τ_dis_ is consistent with our previous results obtained by measuring Kd from binding experiments ([7] and Fig. 1C, *inset*).

From eq. 3 we could potentially evaluate also k_off_^L^. However, recalling that Kd^L^ is larger than Kd^H^ by three orders of magnitude [21], τ_dis_^L^ is expected to be in the millisecond range and thus cannot be measured by our technique as the initial wash time is 2 sec.

The m1 muscarinic receptor, m1R, was also shown to possess voltage sensitivity, but in the opposite direction; the binding of ACh to m1R expressed oocytes is higher at depolarization than at resting potential (*inset* in Fig. 1 and [7]). Corollary, τ_dis_ is expected to be voltage sensitive but in the opposite direction than that of the m2R. Fig. 1D shows that this is indeed the case: τ_dis_ is longer at +5mV than at −88 mV. The results in Fig. 1D further rule out the possibility that the observed effect of membrane potential on the k_off_ reflects an unspecific effect of either membrane potential or K^+^ concentration on the oocytes or the ligand.

### The Deactivation Rate of GIRK Channel (τ_dec_) Reflects k_off_

**Figure 2:**
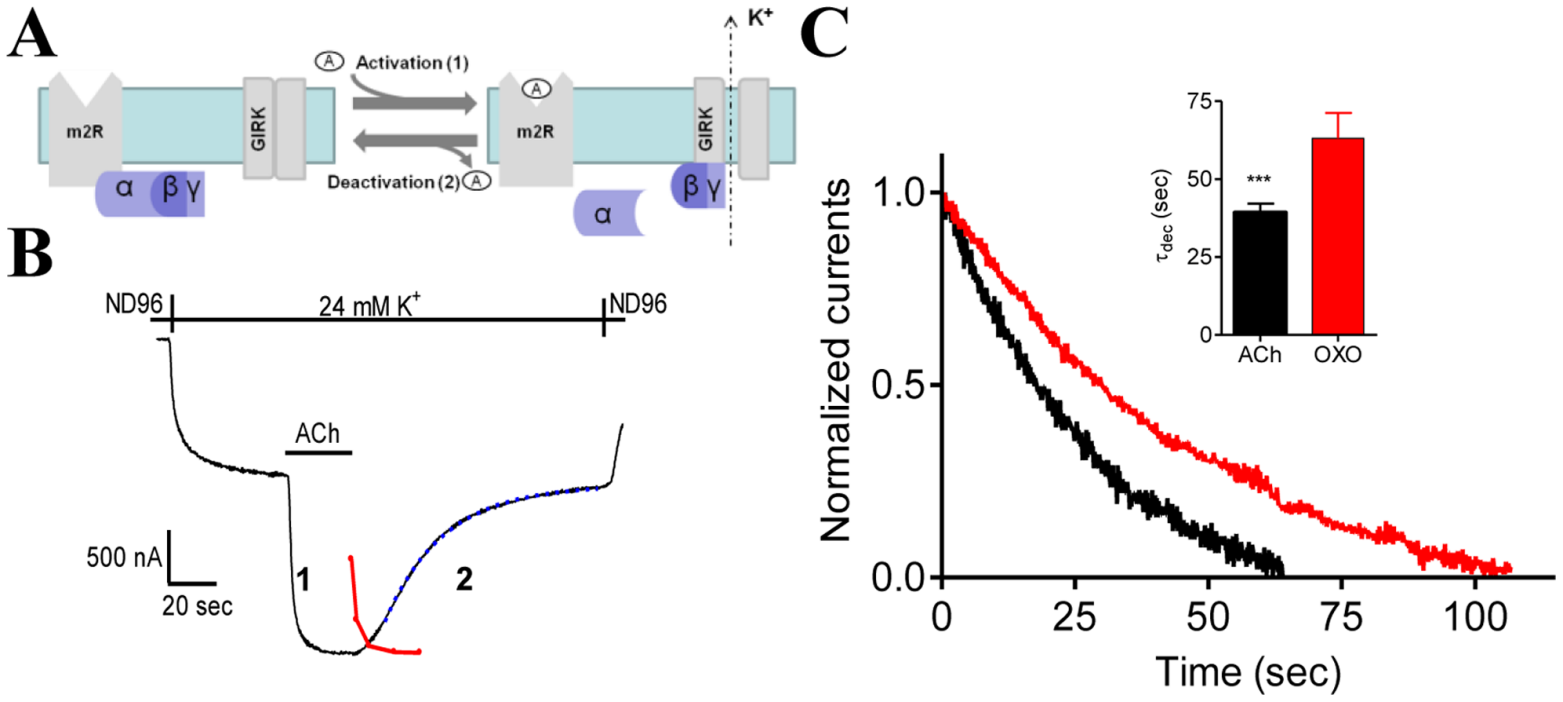
Measurement of τ_dec_ fro the decline of the GIRK current. (A) The functional assay used. Binding of ACh to the m2R leads to G-protein activation. βγ subunits of the activated G-protein then bind to the GIRK channel and open it, inducing I_ACh_. Upon ligand washout, ACh unbinds from the receptor and I_ACh_ deactivates. (B) The basic experimental protocol. The oocyte was clamped at −80 mV and I_ACh_ was evoked by application of 1 µM ACh in 24 mM K^+^ solution (1). Following washout of the ACh with ACh-free solution the current declines (2). The kinetics of the washout, as measured by the disappearance of a colored solution from the bath, is shown as red symbols and line (see text for details) (C) τ_dec_ depends on the affinity of the agonist to the receptor. I_ACh_ decline after wash out faster than GIRK current evoked by OXO. Representative recordings from one oocyte are shown. The currents shown here and in Figs. 3A and 5B, C are normalized to enable comparison of the kinetics of the current decline. *Inset* collected data from 14 oocytes. The two bars are significantly different (p<0.0001).

To corroborate the conclusion that depolarization shifts the m2R from k_off_^H^ to k_off_^L^ in an independent way we turned to electrophysiological measurements of deactivation of the m2R induced GIRK currents (see scheme in Fig. 2A). Whether the time constant of deactivation of the GIRK current (τ_dec_) reflects the dissociation rate of the ligand from the receptor is still under debate. Some studies have shown that, under certain experimental conditions, τ_dec_ reflects the dissociation of the agonist from the receptors [24], [25], while other studies suggested that the hydrolysis of the GTP to GDP determines the deactivation rate of the channel [26], [27]. Hence, we first set to examine which scenario holds true under our experimental conditions.

The basic experimental protocol is depicted in Fig. 2B. An oocyte is voltage clamped at the desired holding potential (−80 mV in this example) in a low-K^+^ (2 mM K^+^) solution, ND96. I_K_ is developed upon replacement of the ND96 by the 24 mM K^+^ solution. Then, ACh (1 µM) was added (Fig. 2B, 1), and I_ACh_ appeared. I_ACh_ was terminated upon washout of ACh (Fig. 2B, 2). The decay of the GIRK current reflects the wash time of ACh+τ_dec_. To evaluate τ_dec_, we first checked the time that is needed to washout ACh from the bath. To do so, a colored solution was applied to the bath and then washed with colorless solution (ND96) and the optical density of samples after different washout times (2–20 sec) was measured. It can be seen (Fig. 2B, red line) that after 5 sec washout the colored solution was almost completely washed out from the bath. Thus, τ_dec_ can be safely estimated from the exponential function fitted to the decay curve (Fig. 2B, blue dashed line) if measured from times longer than 10 sec after the beginning of the washout. We began the fit after the current declined to 80% of its maximal level.

We next examined whether τ_dec_ is determined by the rate of dissociation of the ligand from the receptor, k_off_. Indication that this is the case was provided for the metabotropic glutamate receptor type 3 [12]. There, it was shown that τ_dec_ was shorter for agonist with lower affinity and thus, presumably, higher k_off_. To check whether this is the case also for the m2R we compared τ_dec_ obtained with ACh to that obtained with the muscarinic agonist oxotremorine (OXO) which has higher affinity to the receptor, and therefore presumably lower k_off_ [7]. It is seen (Fig. 2C, collected data is shown in the *inset*) that τ_dec_ of the GIRK current induced by OXO, is larger than τ_dec_ of I_ACh_. This result indicates that under our experimental conditions, assuming that both OXO and ACh are activating the same transduction pathway, τ_dec_ is sensitive to the affinity of the receptor toward its agonist. Because τ_dec_ is evaluated at times where the bathing solution is free from ACh (see Fig. 2B, red line) and thus re-association of ACh to the m2R is practically none existing, τ_dec_ in fact reflects τ_dis_ and hence reflecting the dissociation of a ligand from the receptor.

### Voltage Sensitivity of τ_dec_

Based on the same considerations discussed above for the binding experiments, τ_dec_, as τ_dis_ (see Fig. 1) reflects the distribution of the m2R between high and low affinity states, i.e. k_off_^L^ and k_off_^H^. Thus, we can now examine how voltage affects this distribution using eq. 4
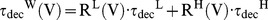
(4)


**Figure 3:**
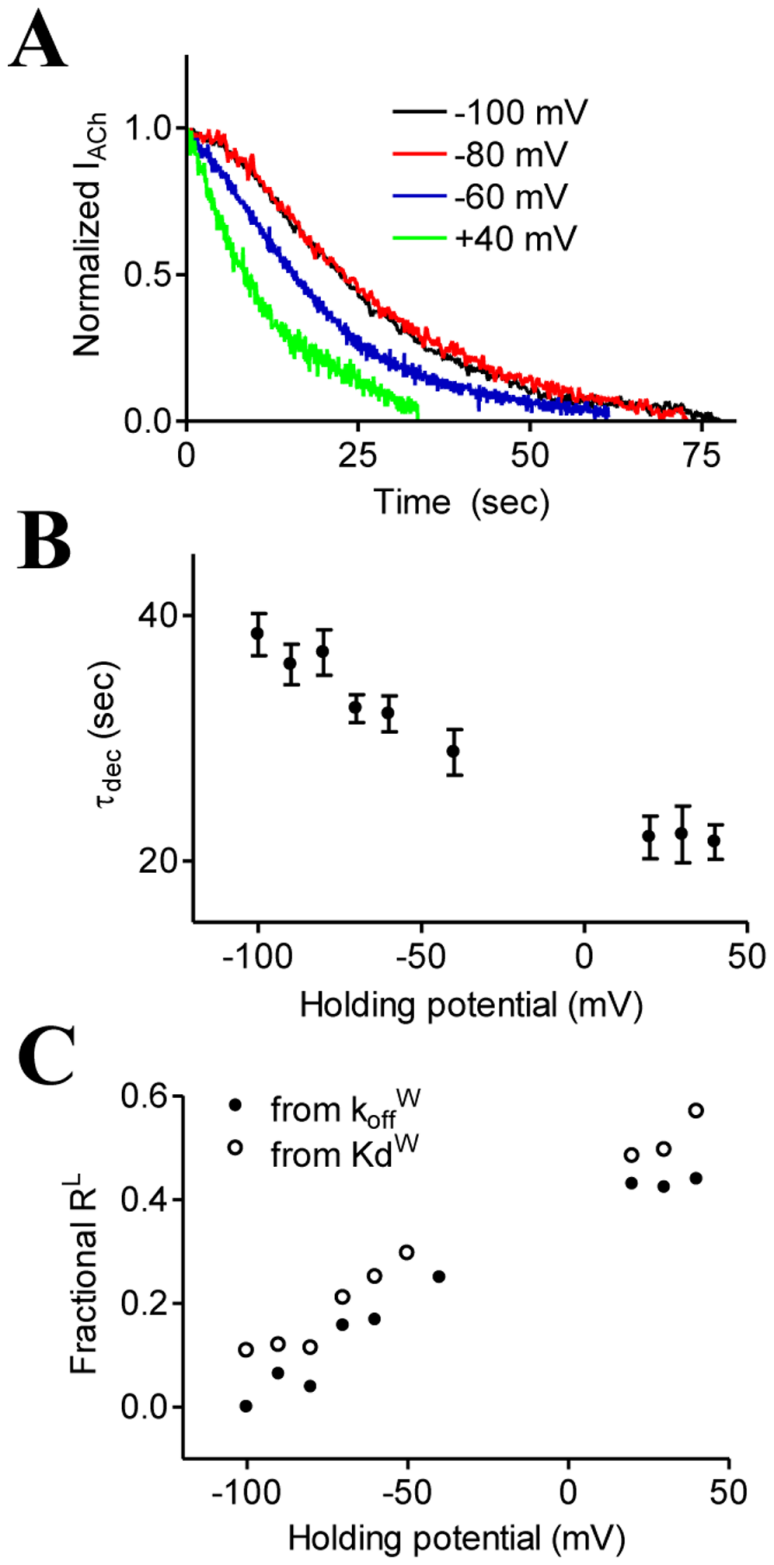
Voltage sensitivity of τ_dec_. (A) The decline of the current evoked by ACh at different holding potentials following washout (beginning at time 0) with agonist free solution. (B) Average± SEM of τ_dec_ at different holding potentials from 8 to 18 oocytes for each holding potential. (C) The dependency of R^L^ in membrane potential, from experimental data taken from [6] (empty symbols) and from the data shown at (B) (filled symbols).

To this end, an oocyte was voltage clamped in one holding potential, and the basic protocol described in Fig. 2B was repeated. After 10 minutes wash in ND96, the holding potential was changed to a different level (selected randomly) and τ_dec_ was measured again employing the same protocol. Usually, measurements were done at 4 or more holding potentials between −100 mV and +50 mV from the same oocyte (Fig. 3A). We were not able to measure adequately τ_dec_ at membrane potentials between −40 mV and +20 mV because of the weak driving force of the GIRK current in this potential range. It is seen (Fig. 3A, B) that, similarly to τ_dis_, also τ_dec_ is highest at resting potential, and decreases as depolarization increases. The decay curves, shown in Fig. 3A were fitted by exponentials from which τ_dec_^W^ was extracted for each holding potential (Fig. 3B). Fig. 3A (black trace) shows that at −100 mV, where most of the receptors are in R^H^, τ_dec_^W^ = 38.4 sec. Similar to the case discussed above for τ_dis_^W^ (eq. 3.1) also for τ_dec_^w^_(−100 mV)_ eq. 4 may be simplified to become
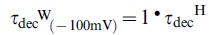
(4.1)


Thus, we conclude that τ_dec_^H^≈38.4 sec. Assuming that, as for τ_dis_, also τ_dec_ = 1/k_off_^H^, we obtain k_off_^H^≈0.026 sec^−1^. The value obtained for k_off_^H^ from the deactivation of the GIRK current is 5 times smaller than that obtained from the direct dissociation measurements. It is possible that in addition to dissociation of the ligand from the m2R, also other downstream processes [26], [27] somewhat contribute to τ_dec_. We believe that in spite of that shortcoming of the functional experimental system, the voltage dependent change in τ_dec_ faithfully reflects depolarization induced shift of R^H^ to R^L^. If this is the case, the distribution of receptors between R^L^ and R^H^ at a given holding potential can be extracted from eq. 4 with τ_dec_^W^ taken from Fig. 3B. To solve eq. 4 we recall that τ_dec_^L^ is much smaller than τ_dec_^H^ (see discussion above and [21]), and can be neglected from eq. 4 as even at +40 mV only ∼60% of the receptor are in R^L^ [7]. Eq. 4 is thus reduced to τ_dec_ = R^H^(V) • τ_dec_^H^. Having τ_dec_^H^, the dependency of R^L^ (1−R^H^) on voltage can be evaluated (Fig. 3C, filled circles). The results correlate well with our previous analysis of this dependency, evaluated by considering the Kd of the m2R (Fig. 3C, empty circles, modified from [6]), reinforcing the conclusion that the difference between R^L^ and R^H^ is implemented by different k_off_ values. As for the binding measurements, τ_dec_^L^ is expected to be in the milliseconds range and cannot be estimated from our measurements of τ_dec_.

### The Mechanism by which Depolarization-induces a Shift between the Two States of k_off_

**Figure 4:**
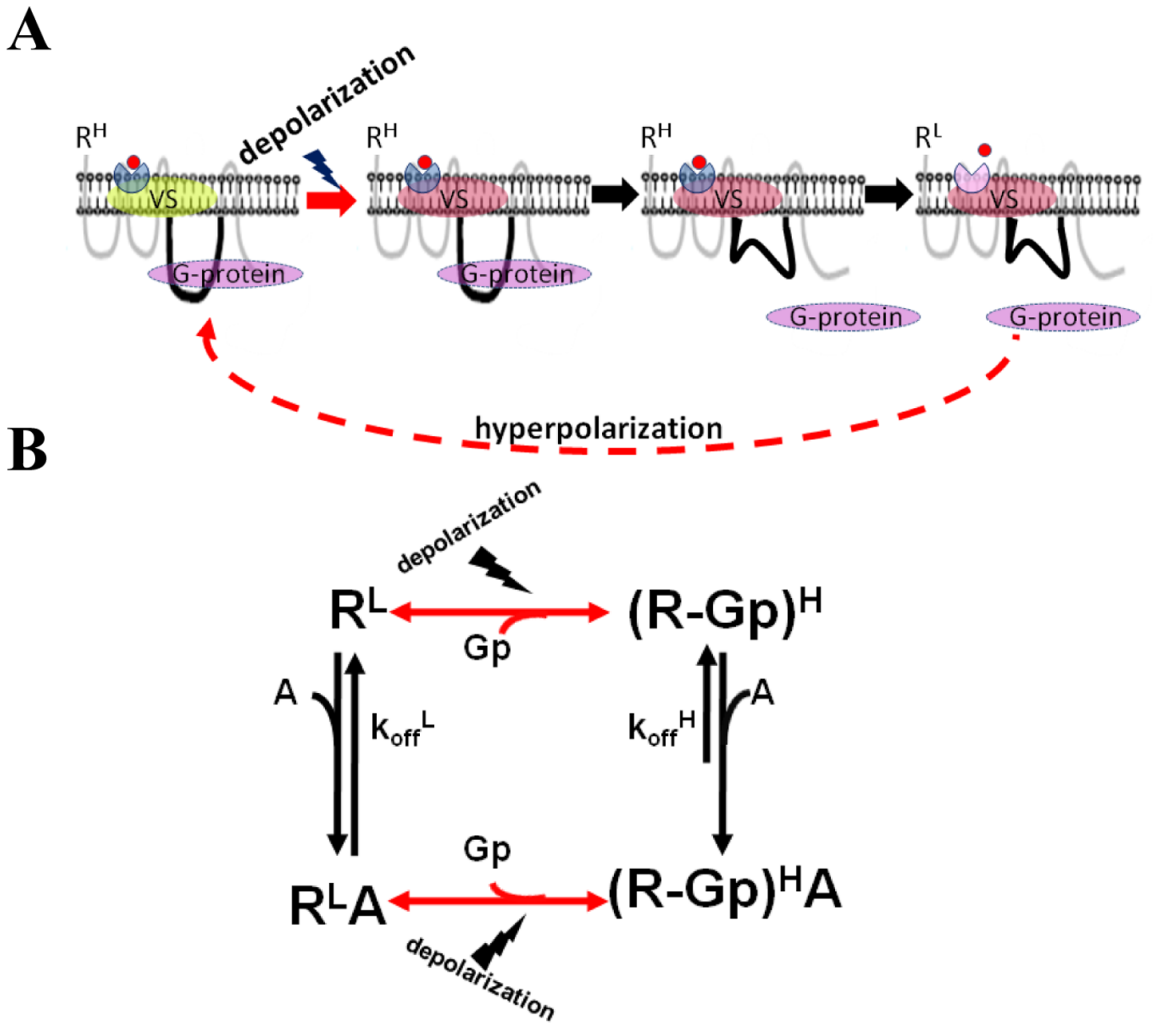
The mechanism proposed to shift receptor between two affinity states. (A) A schematic description. R^H^ is coupled to a G-protein. Depolarization induces charge movement in a putative voltage sensor which induces conformational change in L_3_. This change reduces the probability of the GPCR to couple to its G-protein and that in turn shifts the GPCR into R^L^. Hyperpolarization reverses the process and the GPCR shifts back to R^H^ (dashed arrow). (B) The proposed mechanism for voltage sensitivity in the m2R in the context of the ternary complex model [23]. The GPCR can reside in either R^H^ (bound to G-protein, (R−Gp)^H^)) or in R^L^. Depolarization reduces the likelihood of the receptor to be in R^H^ state (red arrow). When coupled to the G protein, the GPCR acquires k_off_^H^ (low value of k_off_). When uncoupled to the G protein the GPCR acquires k_off_^L^ (high value of k_off_).

We have suggested earlier ([7] and see Fig. 4A here) the following scenario by which depolarization shifts the GPCRs between affinity states: Depolarization induces charge movement in a voltage sensor(s) within the GPCR. This movement is relayed to the third intracellular loop of the receptor (L_3_), a region that is known to be involved in G-protein coupling [28], [29]. The conformational change that occurs in L_3_ reduces (in the m2R) or increases (in the m1R) the probability of the receptor to couple its G-protein. Following the ternary complex model [23], the depolarization induced reduction in the coupling of the m2R to the G-protein leads to increased fraction of receptors that are at low affinity (Fig. 4B). (In a recent study it was suggested that the above scenario does not apply to the α-2 adrenergic receptor [16]).

**Figure 5:**
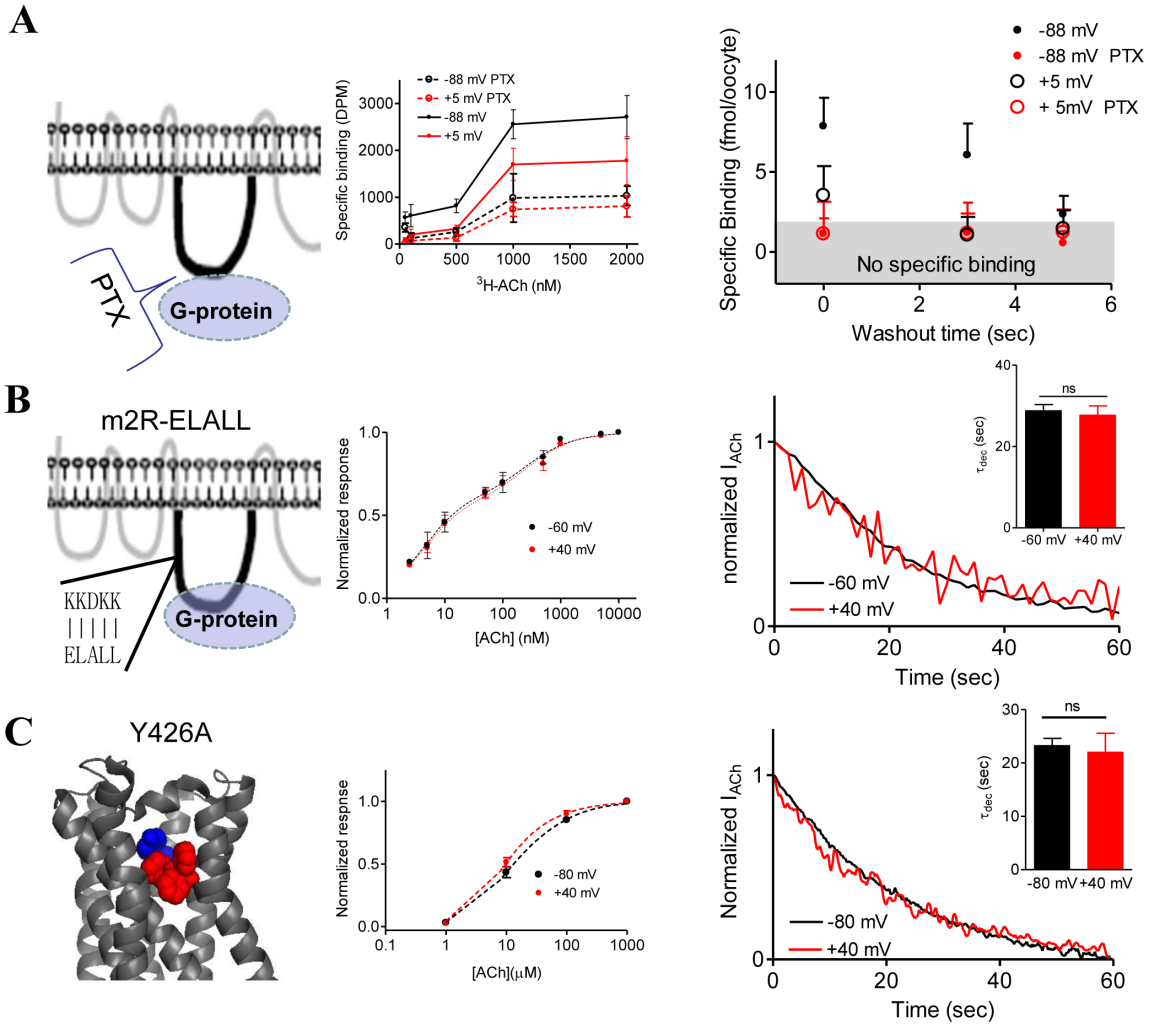
The mechanism of voltage dependent shift in k_off_. (A) Effect of PTX treatment on dissociation of [^3^H]ACh from m2R-expressed oocytes. *Left*, PTX uncouples the G-protein from the GPCR. This causes reduction of the binding affinity and abolishment of the voltage sensitivity of the affinity and abolish the voltage sensitivity of the binding (*Middle*, taken with permission from [7]). *Right*, Dissociation of [^3^H]ACh from m2R-expressed oocytes after different washout times, at two membrane potentials, with and without PTX treatment. The data is average ±SEM of 10 and 12 oocytes. The specific binding after PTX treatment was not significant for all data points. As seen, after PTX treatment the voltage dependence of τ_dis_ was abolished. (B) τ_dec_ is voltage insensitive in m2R- ELALL expressed oocytes. Replacement of 5 residues in the N-terminal of L_3_ (*Left)* abolished the voltage sensitivity of the binding affinity (*Middle*,taken with permission from [6]) *Right*, Measurements of τ_dec_ at −60 mV and +40 mV from the same oocyte. *Inset*. Collected data from 17 (−60 mV) and 23 (+40 mV) oocytes. The bars are not significantly different; *p* = 0.74. (C) τ_dec_ _is_ voltage insensitive in m2R-Y426A expressed oocytes. *Left*, Side view representation of the m2R based on [36] highlighting residue Y426 (red) in the ligand binding site. A ligand bound to the binding site is shown as a blue sphere. *Middle*, Dose-Response relation of the Y426A mutant exhibits low affinity and lack of voltage sensitivity. Taken with permission from [8]. *Right*, τ_dec_ at −80 mV and +40 mV measured from the same oocyte. *Inset*, Collected data obtained with m2R-Y426A expressed oocytes. Each bar represents the mean± SEM of 11 (−80 mV) and 6 (+40 mV) oocytes. The bars are not significantly different (*p* = 0.67).

The scheme in Fig. 4A is based on the following results: (i) Pertussis toxin (PTX), a toxin that uncouples the m2R from its G-protein (Fig. 5A, *left)*, thereby presumably leaving the receptor in its low affinity state [6], [7], abolished the voltage dependence of binding of ACh to the m2R ([7] and Fig. 5A, *middle*). (ii) Mutation in the L_3_, where 5 residues of m2R were replaced by the corresponding residues of the m1R (ELALL, Fig. 5B, *left*) also abolished the voltage dependency of m2R affinity, and the high affinity predominated even at +40 mV ([6] and Fig. 5B, *middle*). (iii) In a recent study [8] it was shown directly that PTX and the ELALL mutant greatly reduced the voltage dependent conformational change in the ligand binding site (detected by fluorescence measurements), and corollary the voltage dependence of m2R binding affinity [8].

The present work enables refining the scheme in Fig. 4. In particular we examine whether treatments that affect the affinity of the m2R did so by affecting the transition between the k_off_ states.

To test whether coupling of the m2R to the G-protein affects k_off_^w^, the experiments described in Fig. 1 were repeated but with oocytes pre-treated with PTX. As mentioned above, treatment with PTX abolished the voltage dependency of [^3^H]ACh binding and the m2R resided in low affinity even at −88 mV. We expect that PTX treatment will also abolish the voltage dependence of τ_dis_ and k_off_^L^ will predominate. Fig. 5A, *right*, shows that this is the case. The specific binding was abolished at both membrane potentials, indicating that the dissociation was completed within the 2 sec of the initial wash. These results are consistent with our previous conclusion [6]–[8] that PTX treatment leaves most of the receptors in R^L^ even at −88 mV, and thus abolishes the voltage sensitivity of the affinity of the m2R.

Next we examined the effect of the ELALL mutant on τ_dec_. It is expected that in this mutant also τ_dec_ will be voltage independent and it will remain in k_off_^H^ even under depolarization. Fig. 5B, *right*, shows that this expectation is met. τ_dec_ was measured as described before (Fig. 3) at two holding potentials of −60 mV and +40 mV. While in wt receptor-expressed oocytes the decay is much slower at −60 mV than at +40 mV (Fig. 3B), in the m2R-ELALL τ_dec_ is voltage independent. (Fig. 5B, *right,* collected results are shown in the *inset*). Note that like the affinity of the m2R-ELALL mutant, which acquired a Kd similar to that measured in −100 mV in wt m2R, τ_dec_ of this mutant at both holding potentials was close to the one measured at −100 mV although did not reach its high value. The voltage independence of τ_dec_ in the ELALL mutant strikingly demonstrates that indeed mutation in the L_3_, a site that is remote from the ligand binding pocket, affects the dissociation of the ligand from the receptor. This observation strongly supports our hypothesis that membrane potential affects the binding affinity of the m2R by altering its coupling to the G-protein.

Both the experiments with PTX and with the ELALL mutant further support our conclusion that depolarization shifts the m2R between two distinct affinity states. This is because treatments that retained the receptor in either high (ELALL mutant) or low (PTX treatment) affinity states, abolished the voltage sensitivity of the m2R.

The final station that eventually determines the affinity of the receptor is its ligand biding site. Indeed, it was shown that mutations in the binding site greatly reduced the conformational change in the ligand binding site and abolished the voltage dependent change in affinity [8]. We thus examined whether such mutations affect the voltage dependent shift of the receptor from k_off_^H^ to k_off_^L^. To this end the experiments described in Fig. 3 were repeated but with m2R mutated in its orthosteric binding site (Y426A, Fig. 5C, *left*), a mutant that was shown to have low affinity toward ACh also at resting potential ([8]. and Fig 5C, *middle*). Fig. 5C, *right* (collected data is shown in the *inset*) shows a recording of the decay of I_ACh_ after ACh washout at two holding potentials. It is seen, that τ_dec_ was voltage insensitive and retained values that are consistent with low fraction of R^H^ even at −80 mV. This is in contrast to the case in wt m2R (Fig. 3B), reinforcing our conclusion that the voltage dependent affinity shift in the m2R is implemented by shift between two k_off_ states.

## Discussion

GPCRs mediate most signal transduction processes. The first step in these processes is binding of an agonist. Recently it was found, for several GPCRs, that the agonist binding affinity is voltage dependent [6]–[8], [12], [15], [21], [30]. This discovery changed our understanding of signal transduction by GPCRs; it depends not only on the agonist but also on membrane potential. In this study we made an important step forward. We show that it is the dissociation of the agonist from the m2R which is voltage dependent.

Signal transduction processes mediated by GPCRs are usually implemented be second messengers and hence are typically slow (with time constants ranging between seconds to minutes). A recent discovery may drastically change this dogma. It was shown [8] that fast depolarization-induced charge movement in the m2R, a prototypical GPCR, underlies the voltage dependence of the binding affinity of this receptor. This finding was crucial in deciphering the mechanism that controls the time course of Ca^2+^-dependent neurotransmitter release. As suggested by the Ca-Voltage hypothesis and validated in numerous publications (reviewed in [31], [32]), the scenario that underlies control of the time course of neurotransmitter release is as follows: At resting potential the relevant GPCR (different for each type of transmitter) resides in R^H^ state, and hence bound to the transmitter even though its concentration in the synaptic cleft is low. The transmitter-bound GPCR physically interacts with the release machinery [33] and keeps release under tonic block. Upon depolarization, two independent events take place. (i) Ca^2+^ channels open to allow Ca^2+^ influx. (ii) Depolarization-induced charge movement causes a shift of the GPCR from R^H^ to R^L^. As a result, the transmitter dissociates, and consequently the tonic block is relived [34] by detachment of the m2R from the release machinery [21] and release initiates. Indeed, charge movement in the m2R was shown to control the extremely rapid process of release of ACh from nerve terminals [35].

Is the shift from k_off_^H^ to k_off_^L^ reported here compatible with controlling such rapid process? Although k_off_^L^ could not be measured in this study, this parameter may be estimated from earlier studies. Measurement of the affinity of the m2R in nerve terminals (synaptosomes) yielded Kd^H^ of ∼20 nM and Kd^L^ of ∼100 µM [21]. Assuming a constant k_on_ value of ∼10^7^ M^−1^•sec^−1^, we obtain k_off_^H^ of 0.2 sec^−1^ and k_off_^L^ of 1000 sec^−1^. Such a short dissociation time constant of the low affinity state (τ_dis_^L^ = 1 msec) may enable the depolarization induced shift of the m2R from R^H^ to R^L^ to guarantee the brief time course of neurotransmitter release. A similar effect on the k_on_ would have a delayed influence on the occupancy of the receptor and thus is less likely to play a role in the tight control of the time course of release. Note, that the value of k_off_^H^ evaluated here from the binding experiments is similar to that reported before in a more physiologically-relevant preparation (synaptosomes, [21]), thus indicating that the conclusions from this study may be physiologically relevant even though they were obtained using a heterologous expression system.

Finally, the present study also provides new insights on the mechanism by which voltage affects the affinity of the m2R. Two hypotheses were suggested: (i) Membrane potential affects directly the receptor’s binding site and thereby affects its agonist binding affinity [11], [13], [15]. This hypothesis is based on the findings that for a given receptor, depolarization reduces the affinity of some agonist, while it increases or has no effect on other agonists. (ii) We suggest that voltage affects the coupling between the receptor and the G protein and in turn the binding affinity. The experimental results that support hypothesis (ii) were described before ([6]–[8] and the Results section here). Briefly, PTX reduced both the depolarization induced conformational change in the binding site of the m2R (detected by fluorescence measurements) and concomitantly reduced the binding itself and abolished the voltage dependence of binding. Direct experiments, similar to those done to test hypothesis (ii), still need to be done to test hypothesis (i) described above.

Interestingly, the mechanism suggested in hypothesis (ii) does not require introduction of a new mechanism of affinity regulation. Rather, voltage regulates a fundamental and a well established mechanism that determines the affinity of GPCRs. Specifically, when a GPCR is coupled to its G-protein it resides in a high affinity (i.e., according to our novel finding here, low k_off_) and when uncoupled it resides in a low affinity state (i.e. high k_off_), and voltage regulates the coupling of the GPCR to its G protein.
